# Toxicological Aspects, Safety Assessment, and Green Toxicology of Silver Nanoparticles (AgNPs)—Critical Review: State of the Art

**DOI:** 10.3390/ijms24065133

**Published:** 2023-03-07

**Authors:** Maciej Noga, Justyna Milan, Adrian Frydrych, Kamil Jurowski

**Affiliations:** 1 Department of Regulatory and Forensic Toxicology, Institute of Medical Expertise, Łódź, ul. Aleksandrowska 67/93, 91-205 Łódź, Poland; 2 Laboratory of Innovative Toxicological Research and Analyses, Institute of Medical Studies, Medical College, Rzeszów University, Al. mjr. W. Kopisto 2a, 35-959 Rzeszów, Poland

**Keywords:** nanotechnology, toxicology, silver nanoparticles, green synthesis, nanoparticle toxicity

## Abstract

In recent years, research on silver nanoparticles (AgNPs) has attracted considerable interest among scientists because of, among other things, their alternative application to well-known medical agents with antibacterial properties. The size of the silver nanoparticles ranges from 1 to 100 nm. In this paper, we review the progress of research on AgNPs with respect to the synthesis, applications, and toxicological safety of AgNPs, and the issue of in vivo and in vitro research on silver nanoparticles. AgNPs’ synthesis methods include physical, chemical, and biological routes, as well as “green synthesis”. The content of this article covers issues related to the disadvantages of physical and chemical methods, which are expensive and can also have toxicity. This review pays special attention to AgNP biosafety concerns, such as potential toxicity to cells, tissues, and organs.

## 1. Introduction

Currently, scientists and consumers are increasingly willing and intensively looking for alternative solutions in the context of improving or regaining health. They often look for therapeutic solutions used in previous years, whose usage is often now required by resistance to the practise, to microorganisms to modern treatments [1]. The development of Evidence-Based Medicine has led to the exclusion of certain substances or a change in the way they are used.

The term “nanosilver” in the popular science literature often refers to various types of nanoparticles (Ag Nanoparticles, AgNPs) and is used interchangeably with the term “colloidal silver”. Silver nanoparticles range in size from 1 to 100 nm and have a large surface area. Large differences in diameter translate into the biological properties of individual particles. This characteristic is influenced by the surface charge, which determines the toxicity [2]. An important determinant of the toxic effect of nanosilver is the route of administration to the human body, the concentration and duration of administration, and the bioavailability of the particles and accumulation, as well as their size, distribution in tissues, penetration, and cellular absorption [3]. 

Nanosilver is an example of a substance whose application is changing dramatically and causing concern in terms of its negative impact on human health [4]. A growing number of consumer items are using AgNPs because of their special characteristics. AgNPs are widely used, increasing human exposure to them through a variety of channels [5]. There are few studies on the toxicity of such silver, and their results are often contradictory [6]. The hope of improving the safety of their use is brought about by nanosilver particles obtained from natural ingredients (so-called green synthesis) [7]. Nanometal solutions (e.g., silver, gold, and copper) are very popular among users of Internet forums, which confirms the interest in these type of substances. In online auctions, they appear under various names, including colloidal silver and nanosilver. Interest in silver nanoparticles has increased significantly since the beginning of the COVID-19 pandemic. This has translated into an increase in the intensity of research on nanoparticles in various scientific areas [8]. Synthesis: Of the several methods used to produce nanosilver, the so-called green synthesis is one that is becoming increasingly significant [7]. Despite the many beneficial effects of nanosilver in microbiological, health, and consumer spheres, it is not classified in the European Regulation on Chemical Substances (REACH) [9]. Taking into account all of the arguments presented above, the topic seems to be extremely important and warrants further research. Numerous studies have been conducted; however, not every study does so in a thorough and holistic way. An interesting aspect that favours considering the use of nanoparticles is their production and application through “green synthesis”. This review details the safety aspects and application of the green AgNPs’ synthesis.

## 2. Materials and Methods

### 2.1. Search for AgNP Publications on Toxicological Aspects, Safety Assessment, and Green Toxicology of Silver Nanoparticles Data

Scopus, Google Scholar, Web of Science, and PubMed are used for the critical analysis of key elements in the context of AgNPs with regard to toxicological issues, safety assessment, and green toxicology of the silver nanoparticle data. It should be emphasised that both the “grey” literature (e.g., Internet forums) and the aforementioned scientific sources were searched during the data collection process. The primary terms AgNPs, nanoparticles, green synthesis, natural nanoparticle, natural metallic nanoparticle, nanotechnology, and nanoparticle toxicity were combined in various ways. The selection of the studies was made in two steps: (1) selection of titles and abstracts and (2) analysis of the full text. Each author independently and at various times chose the title and abstract. After the problem’s formulation was defined, flawed and illogical analyses were critically identified. Tests that met the capability criteria for qualification were diverted to the next stage of the round-robin process. Research studies that were not related to the problem formulation or did not meet the validity criteria were rejected. We analysed all available sources (*n* = 207 articles and related texts—year of publication from 2000 to 2023). Only articles or studies related to the presence or function of AgNP in terms of toxicology were considered to narrow down the sources. The authors then conducted a thorough analysis of the entire text, taking into account all the problematic recopies.

### 2.2. Classification and Presentation of the Results

To focus on sources, only papers or research that discuss the existence or role of AgNPs (e.g., in vitro/in vivo toxicology studies on AgNPs, toxicity of AgNPs against immune cells, toxicity of AgNPs against normal human cell lines, unfavourable effects of AgNPs, organ toxicity of AgNPs, toxicity mechanisms, non-oxidative stress-related mechanisms, and complex toxicity evaluation of AgNPs) are taken into account. We provide information in three paragraphs for each aspect to ensure proper readability:

(1) Toxicological aspects of silver nanoparticles;
(2) Safety assessment of silver nanoparticles in cosmetic products;
(3) Green toxicology of silver nanoparticles.

## 3. Toxicological Aspects of Silver Nanoparticles

### 3.1. In Vitro Toxicology Studies on AgNPs

Nanotechnology has rapidly grown with utilisation in a wide range of commercial products throughout the world. However, there is still a lack of information on the increase in human, animal, and ecological exposure to nanoparticles, including AgNPs, and the possible risks related to their short-term and long-term toxicity. This section provides an overview of the possible risks and toxic effects of AgNPs.

In vitro studies, in addition to demonstrating the positive properties of AgNPs, such as antimicrobial and antifungal activity, have also revealed their adverse and toxic health effects on cells or bacteria after exposure to these nanoparticles. Experiments with toxicology studies involve subjecting a number of cells and organs to different doses of chemicals, and their response is taken into account over a given period of time [1]. Reactions are dose-dependent; based on these reactions, the appropriate dose of drug administered can be determined, along with the exposure limit to avoid side effects, median toxicity (MD_50_), and median lethal dose (LD_50_) [2]. In traditional cytotoxic assays, the emphasis is mainly on soluble chemicals that, upon administration, present cellular toxicity [3]. In the case of nanoparticles, this is determined on the basis of specific sizes, shapes, and their densities. This results in the aggregation and agglomeration of nanoparticles at specific sites in target cells or organs by diffusion across membranes, resulting in a colorimetric result. Consequently, traditional in vitro assays on nanoparticles lead to a misinterpretation of the cellular uptake data, making the results less reliable [4]. The cytotoxic effect of silver nanoparticles has been characterised mainly in terms of oxidative stress and genotoxic effects.

The production of reactive oxygen species (ROS) stimulated by the cell uptake of AgNPs causes oxidative stress and genotoxic effects. Increased ROS production, in large numbers, induces cell death through apoptosis or necrosis [5]. AgNPs at a dose of 50 μg/mL affected direct DNA damage in mouse embryonic cells and fibroblasts. The effect was indirectly measured by increased expression of DNA repair proteins (Rad51 and H2AX) and upregulation of p53 (cell cycle checkpoint protein) [6]. AgNP action on murine embryonic fibroblast cells (NIH 3T3) resulted in upregulation of hemeoxygenase 1 (HO-1) expression, ROS induction, autophagy, and apoptosis [7]. AgNPs at doses greater than 1 µg/mL induced cytotoxicity and abnormal cell morphology in the human hepatoma cell line (HepG2) [8]. A dose of AgNPs (0.7 μg/mL) in human hepatoma cells caused upregulation of superoxide dismutase 1 gene expression [9]. The toxicity of AgNPs depends both on size and shape; a study with alveolar macrophages showed that silver nanoparticles with an average size of 15 nm induced the greatest loss of mitochondrial activity [5]. In human umbilical vein endothelial cells (HUVEC), silver nanoparticles severely induced apoptosis, inhibited proliferation, and damaged the cell membrane [10]. AgNPs induced cytotoxicity in baby hamster kidney (BHK21) and human colon adenocarcinoma (HT29) cells in vitro; the effect of AgNP, at a concentration of 11 µg ml^−1^, was tested by progressive nuclear double AO/EB staining of treated cell nuclei at different times, then confocal microscopy images of dual stained cells demonstrated that the live cell nuclei stained green due to AO uptake and that their numbers rapidly reduced over time due to increased cell death. The same image shows that EB gradually is taken up by the nuclei as a result of cell membrane perforation caused by apoptosis, which dyed the nuclei red. This effect became more pronounced after 4 h. After 6 h, images of treated and untreated cells show the presence of fragmented or condensed chromatin made up of apoptotic nuclei in treated cells, and well-organised chromatin structures in untreated living cells. As a result, the nuclear staining experiment demonstrates that apoptosis began 4 to 6 h after AgNPs were added to the culture media [11]. The reduction in glutathione level and damage to the cell membrane in HeLa cell lines was due to exposure to AgNPs in a dose of 100 μg/mL [12]. AgNPs reduced albumin release, which had an impact on hepatocyte homeostasis, and ultimately proved to be highly cytotoxic [13]. The high concentration of AgNP (333 μM) caused long-term growth inhibition in human keratinocyte cells (HaCaT) [14]. In the case of human lung fibroblasts (IMR-90) and human glioblastoma cells (U251), silver nanoparticles damage DNA indirectly through increased ROS production and reduction in ATP production (related to mitochondrial damage), which weakens energy-dependent DNA repair mechanisms [15]. Silver nanoparticles induce oxidative stress, which is an important factor in their genotoxic activity in BEAS-2B cells [16]. Exposure to high doses of AgNP (100 μg/mL) for 24 h of coronary endothelial cells in rats resulted in increased nitric oxide production. This increased cell proliferation, while low dose (<10 μg/mL) resulted in a decrease in mitochondrial function. Nitric oxide plays an important role in the cardiovascular system, suggesting a different direction for the biological effects of AgNP toxicity [16,17]. In *Drosophila melanogaster*, induction of oxidative stress and upregulation of the expression of the heat shock protein (HSP 70) expression were caused by exposure to silver nanoparticles at doses of 50–100 μg/mL for 24–48 h [17,18].

AgNPs have also been shown to be detrimental to bacteria in in vitro experiments, with these studies including simple terrestrial bacteria, as well as compiled aquatic bacteria [18]. In *Nitrosomonas europaea*, AgNPs at a dose of 2.5 μg/L cause upregulation of ammonia monooxygenase genes expression [19]. The authors of [20] showed efficacy in antibacterial activity against *Lactobacillus Acidophilus pneumoniae* in the presence of AgNPs at a concentration of 20–100 mg/L. The antimicrobial properties of the silver nanoparticles depend on their size. The small size of the silver nanoparticles had an increased active surface that reacts with bacterial cells, which increased the number of extract molecules anchored to the surface of AgNPs [21]. At high doses of AgNPs (12.5–100 μg/mL), the growth rate of *Escherichia coli* decreased [22]. On the other hand, exposure to silver nanoparticles at a dose of 1.35 μg/mL caused a slight increase in the growth rate of *Escherichia coli* [23]. *Pulicaria glutinosa* amplifies the solubility of AgNPs (50–500 μg/mL). This results in increased toxicity for the following microorganisms: *Pseudomonas aeruginosa*, *Staphylococcus aureus*, *Escherichia coli*, and *Micrococcus luteus* [24].

In general, in vitro studies have shown adverse health effects of cells or bacteria after exposure to AgNPs. The mechanism of AgNP-dependent cytotoxicity in in vitro assays is primarily based on the induction of reactive oxygen species (ROS). Cytotoxicity and genotoxicity of AgNPs depend mainly on the size, concentration, and duration of exposure. Exposure to silver nanoparticles causes a decrease in GSH levels (glutathione), lipid peroxidation, increased expression of ROS-responsive genes, and an increased level of their proteins, which in turn leads to DNA damage, apoptosis, and necrosis [25].

The study of cytotoxicity in vitro in cell culture of molecules with therapeutic potential is an excellent introduction to generally understood toxicity research. In vivo testing verifies the efficacy and safety of drug candidates prior to human testing.

### 3.2. In Vivo Toxicology Studies on AgNPs

Compared to in vitro studies, much less information is available on the possible mechanisms of AgNP toxicity from in vivo studies. Existing in vivo studies on the cytotoxicity and genotoxicity of AgNPs answer the question of the actual toxic effect of silver nanoparticles on many species, including terrestrial invertebrates, vertebrates, aquatic organisms, and higher plants. As a result of their very small size, AgNPs have great mobility in various environments. Therefore, living organisms are easily exposed to nanoparticles through pathways such as inhalation, ingestion, and the skin. AgNPs can travel from exposure to other vital organs and enter cells [4]. Silver nanoparticles can cause defects in the spinal cord, heart, and eye [1]. Exposure to AgNPs have caused innumerable toxicological reactions, including the following: cardiovascular system, respiratory system, central nervous system, liver tissue, and skin tissue effects following local administration of silver nanoparticles. These effects are discussed below.

Exposure to AgNP (60 nm) by ingestion was tested in Sprague–Dawley rats. After 28 days, there were no significant changes in body weights from the AgNP dose in both male and female rats. The only noticeable changes were the values of alkaline phosphatase and cholesterol, with a dose of 300 mg AgNP that damaged the liver of rats [26]. Prolonged inhalation exposure in Sprague–Dawley rats showed that the lungs are the main target tissues affected by AgNP [27]. Administration of AgNPs up to 20 mg/kg/day does not cause toxicity during gestation of rats. In contrast, prenatal exposure increases Ag levels in the tissues of their offspring [28]. AgNPs caused damage to the rat liver by dysregulation of lipid metabolism when exposed to 500 mg/kg/day for 81 days. The liver and heart were highlighted as the most sensitive organs to the damaging effects of silver nanoparticles [29]. Exposure to AgNPs for 10 days resulted in cytotoxicity or minimal pulmonary inflammation in mice [30]. The potential neurotoxicity and immunotoxicity related to exposure to AgNPs were investigated in C57BL/6 mice. Silver nanoparticles influenced the modulation of gene expression related to motor neurone disorders, neurodegenerative diseases, and immune cell function [31]. There are also toxicological studies related to organism exposure to AgNPs by skin exposure or injection [31,32]. For organisms such as crustaceans, fish, and protozoa, silver nanoparticles showed LC_50_ values below 10 mg/L [33]. The acute effects of AgNPs’ toxicity in three *Daphnia* species occurred at doses of 121 μg/L, 0.95 μg/L, and 13.9 μg/L for *D. magna*, *D. pulex,* and *D. galeata*, respectively [34]. Mortality (LC_50_) of *Daphnia magna* was observed among three surface coatings of AgNP: lactate (28.7 μg/L), polyvinylpyrrolidone (2.0 μg/L), and sodium dodecylbenzene sulphonate (1.1 μg/L). In vivo studies focused on the physicochemical properties of nanomaterials with their toxic reactions [35]. AgNP size-dependent toxicity affects *Eisenia fetida* [36]. AgNPs showed toxicity that was dependent on the surface charge of the tested *Bacillus* species. In that study, different types of silver nanoparticles were considered: uncoated H2-AgNPs, citrate-coated AgNPs, polyvinylpyrrolidone-coated AgNPs, and branched polyethyleneimine-coated AgNPs [37]. The influence of various concentrations and sizes of AgNPs on seed germination and growth of jasmine rice seedlings were investigated. With an increase in the concentration of silver nanoparticles, the level of seed germination and seedling growth decreased [38]. The effect of AgNPs on roots at a dose of 50 and 75 ppm caused oxidative damage and strongly reduced root growth [39]. The in vivo study of AgNPs allows us to better assess the acute and chronic systemic toxicity of nanomaterials. The silver nanoparticles resulted in a reduction in the hatch rate of zebrafish embryos. Furthermore, the zebrafish larvae had an abnormal dorsal chord, damaged eyes, and a curved tail. AgNPs were distributed throughout the brain, heart, yolk, and blood of zebrafish embryos. Furthermore, silver nanoparticles caused apoptosis in zebrafish embryos [40]. Silver nanoparticles of smaller sizes (20 nm) compared to their large counterparts (100 nm) were more toxic in the zebrafish embryo [40,41]. After exposure to AgNPs, the zebrafish showed behavioural changes [41,42]. In the zebrafish and algae species tested, silver nanoparticles caused acute toxicity at a dose of 40 μg/L for 48 h [43]. AgNPs were cytotoxic to rainbow trout cell lines and their hepatocytes [44]. Exposure to dose-dependent AgNPs in catfish embryos caused mortality, malformations, and DNA fragmentation [44,45].

This review indicates that the most common causes of AgNPs-induced toxicity include oxidative stress, DNA damage, and apoptosis. An in vivo study showed that exposure to silver nanoparticles triggers effects in various major organs [45,46]. Exposure to silver nanoparticles causes reproductive disorders, malformations, and morphological deformations in many non-mammalian animal models [25]. Most of the available information on the mechanisms of the toxicity and related effects of AgNP comes from in vitro studies, and only limited information comes from in vivo studies [4].

### 3.3. Toxicity of AgNPs against Immune Cells

AgNPs are the most widely used nanoparticles in consumer products. However, their toxicity has raised concerns that limit their use. This section of our review presents the toxicity of AgNPs on immune cells. The physicochemical properties of nanoparticles, such as size, distribution, crystallinity, surface charge, surface coating, synthesis methods, and reactivity, are one of the key factors affecting the immune response [47]. Modification of the surface of the nanoparticles seems to be of greatest relevance to the immune system. The outer coating determines the toxicity of nanoparticles [48]. Silver nanoparticles have stimulating and inhibitory effects on cytokine production associated with the inflammatory response and are likely to depend on cell type and dose [3]. Silver nanoparticles can modulate cytokines involved in wound healing [49]. Interactions between nanoparticles and the innate immune system can affect the adaptive immune response through the production of cytokines and chemokines. IL-1β, a crucial cytokine involved in lymphocyte activation and proliferation, is produced by monocytes in response to exposure to AgNPs. The decreased amount of IL-1β may be related to the impairment of the innate immune response caused by AgNPs [50]. On the other hand, in human epidermal cells, the result was an increased number of IL-1β, IL-6, IL-8, and TNF-α [32]. The expression of the genes TLR2, Myd88, IL-8, NF-κB, and IL-1β was downregulated with increasing concentration of silver nanoparticles [50]. Silver nanoparticles have a toxic effect on the proliferation and expression of human lymphocyte cells and peripheral blood mononuclear cells (PBMCs) [51]. Primary human blood mononuclear cells were exposed to AgNPs with a particle size of 5 nm and 28 nm. Based on the measurement of IL-1β and the induction of inflammatory body formation, it turned out that smaller particles have a greater potential to activate innate immunity [52]. Furthermore, smaller particles also caused higher cytotoxicity in monocytes and macrophages than larger particles [52,53]. In addition, small AgNPs (10 nm) have toxic effects on human blood mononuclear cells, and their toxicity is both time- and dose-dependent [53,54]. AgNPs, in a concentration-dependent manner, induce cytotoxicity and inhibit cytokine proliferation and production, including IL-5, INF-γ, and TNF-α in peripheral blood mononuclear cells. Furthermore, silver nanoparticles can accumulate in the immune organs and affect the number of immune cells and the production of cytokines [55]. Exposure to silver nanoparticles in isolated monocytic THP-1 cells and peripheral blood mononuclear cells (PBMC) causes significant immunotoxicity. In both PBMC and THP-1 cells, the internalisation of AgNPs’ results in increased expression of Myd88, MEKK1, and early regulation of oxidative stress genes [56]. AgNPs internalised into the cytosol and nucleus of human THP-1 monocytes induce monocytic cell death by degradation of the stress sensor ATF-6 and activation of the NLRP-3 inflammasome [56,57]. AgNPs showed high dose-dependent immunomodulation of T cells and monocytes [58]. The immunomodulatory activity of AgNPs varies depending on the type of immune cell and the stage of differentiation. In the example of differentiation of human promyelocytic leukaemia cells (HL-60) into granulocytes or macrophage-like cells, and differentiation of human monocytic cells (U-937) into monocytes and macrophages, differentiated cells were found to exhibit greater resistance to AgNP-induced cell death than undifferentiated cells [58,59]. Alveolar macrophages responded with increased pro-inflammatory mediator production of pro-inflammatory mediators (TNF-α, MIP-2, and IL-1β) after exposure to AgNP [5]. The effect of human macrophages on concentrations of 5 nm or 100 nm silver nanoparticles showed that smaller nanoparticles induced stronger expression of pro-inflammatory cytokines (IL-8) and stress genes (hemeoxygenase-1 and heat shock protein-70) than exposure to 100 nm AgNP [60]. MAPK and NF-κB pathways, which lead to the transcription of many genes involved in inflammatory responses and induce deleterious inflammatory responses, can be activated by AgNP [60,61]. AgNPs exhibit independent immunomodulatory activity based on cytotoxicity by inducing NF-kB activation and the resulting triggering of pro-inflammatory genes, including IL-6 and IL-1β, by macrophages RAW264.7 after exposure to high concentrations (10 mg/mL) of silver nanoparticles [62]. In the case of AgNPs, exposure to these metallic nanoparticles can affect the immune system directly or indirectly. For example, exposure to AgNPs induces inflammation and releases chemokines by activating neutrophils [63]. Human neutrophils are modulated by silver nanoparticles. AgNPs interact with neutrophil cell membranes and infiltrate cells, and are located in vacuole-like structures distributed throughout the cytosol. Internalisation of AgNP increases the rate of neutrophil apoptosis and inhibits de novo protein synthesis [63,64]. Internalised AgNPs are known to increase oxidative stress, thus enhancing the production of reactive oxygen species in human neutrophils [64,65]. The effects of AgNPs on neutrophils include nanoparticles triggering the release of extracellular neutrophil traps and inhibiting the formation of nitric oxide, inhibiting the activity of protein phosphatase, and causing increased intracellular levels of reactive oxygen species [66]. Furthermore, human mesenchymal stem cells showed a decrease in pro-inflammatory factors (IL-6 and IL-8) after exposure to AgNPs. When cells were exposed to less than 5 µg/mL of AgNPs, an increase in the pro-inflammatory factor (IL-8) was observed [67].

### 3.4. Toxicity of AgNPs against Normal Human Cell Lines

#### 3.4.1. Nervous System

The effect of silver nanoparticles on human neural stem cells (NSCs) by increasing mitochondrial production of reactive oxygen species led to apoptosis and necrosis of NSCs [68]. In addition, silver nanoparticles negatively affected mature neurones by triggering abnormalities in cytoskeleton formation, presynaptic and postsynaptic proteins, and mitochondrial function, leading to cell death [68,69]. Mouse neural cells exposed to AgNPs induced the secretion of pro-inflammatory cytokines and the deposition of amyloid beta (Aβ) [70]. The toxic effects of AgNPs have been observed in human neuronal SH-SY5Y cells and human glial D384 cells at low doses (0.5 μg/mL) and short-term (4–48 h, 1–100 μg/mL) or long-term (up to 10 days, 0.5–50 μg/mL) exposure [71]. In vitro studies of the effects of AgNP on neurones and astrocytes derived from embryonic stem cells (ESCs) have shown that exposure to nanoparticles reduces the expression of postsynaptic proteins, changes the morphology of astrocytes, causes neurodegeneration, and reduces the growth of neurites [72].

#### 3.4.2. Digestive System

After oral exposure, AgNPs are absorbed through the gastrointestinal tract, nanoparticles enter the blood, and thus into the organs [73]. Consistent oral administration leads to organ toxicity and inflammatory responses. Smaller silver nanoparticles accumulate in organs such as the brain, lungs, liver, kidneys, and testes [74]. Exposure to AgNPs in the liver of the offspring induces oxidative stress and apoptosis [75]. Treatment with AgNPs by oral route have altered the function of the small intestine mucosa due to the devastation of the microvilli. Therefore, absorption through the intestinal epithelium has been reduced, leading to weight loss in mice [76]. AgNPs are toxic in mice in vivo because they induce changes in the architecture of histological sections of the liver such as vacuolisation and swelling of hepatocytes, oedema around the blood vessel, and induce apoptosis [76,77]. Silver nanoparticles have been proven to penetrate the cell membrane and penetrate the mitochondria. This causes oxidative stress, inflammation, and leads to apoptosis during incubation with human gingival fibroblast cells [78].

#### 3.4.3. Respiratory System

Regarding genotoxicity, in normal human lung fibroblast cells (IMR-90), exposure to AgNPs indirectly induced ROS production or decreased ATP production, resulting in aberrations of the chromosomes and altering energy-dependent DNA repair mechanisms [15]. The toxicology research of AgNPs-treated lung cell line showed that small (10 nm) silver nanoparticles compared to larger sized were more toxic [79]. In another study on human lung epithelial cells (A549), exposure to 56 nm size AgNPs caused upregulation of pro-inflammatory cytokines (IL-1β and IL-6) [80]. Furthermore, silver nanoparticles induced genes responsible for cell cycle progression and therefore caused chromosomal damage, cell cycle arrest, and cell death in human BEAS-2B cells [81].

#### 3.4.4. Cardiovascular System

A similar effect on the cardiovascular system was observed in Hartley albino guinea pigs. Silver nanoparticles smaller than 100 nm, with a dose of 100 ppm, caused cardiomyocyte deformities, congestion, inflammation, and haemorrhage [4]. Exposure to AgNPs affected the cardiovascular system in zebrafish. Nanoparticles caused abnormal heart morphology, cardiac arrhythmias, circulatory defects, pericardial oedema, and slow blood flow [82].

#### 3.4.5. Urinary System

Cytotoxic effects have been observed in kidney cell lines after exposure to high doses of AgNPs. The intraorganic accumulation of nanoparticles at higher doses may cause toxicity in vivo. At low doses, no cytotoxic effects were observed in kidney cell lines in vitro [83]. Silver nanoparticles synthesised with the use of *Rhizophora apiculata* extract were found to be nanotoxic to HEK-293 cells (human embryonic kidney) cells, and cell inhibition effects were dose-dependent [83,84].

#### 3.4.6. Sensory Organs

Retinal cells absorbed low concentrations of AgNPs, causing oxidative stress in an increasing number of cells, which disrupted their primary structure [85]. The toxicity of silver nanoparticles in the rat ear model manifests itself through mitochondrial dysfunction leading to hearing loss, either permanent or temporary depending on the dose [86].

#### 3.4.7. Reproductive System

The survival or death of embryonic stem cells caused by exposure to AgNP is primarily due to the induction of autophagy leading to apoptosis [7]. In germ cell lines, dependence on the size of the silver nanoparticles is the main toxicity criterion. For example, spermatogonal stem cells were resistant to AgNPs of a larger size compared to smaller AgNPs [87]. The internalisation of AgNPs by mouse sperm disrupted the development of the embryo through reduced fertilisation of the oocyte. Furthermore, mortality induced by oxidative stress, as well as mitochondrial copy numbers and morphological abnormalities, also increased [88].

### 3.5. Unfavourable Effects of AgNPs

Silver nanoparticles (AgNPs) are one of the nanomaterials that most of us have already come into contact with because they are used in various types of commercially available products [89]. They are already widely used around the world, but we lack full knowledge of their toxic effects or their safety [90]; therefore, it is necessary to accurately estimate the adverse effects of AgNPs and to understand the health risks associated with the interaction of silver nanoparticles with the human body and ecosystem [91]. The increasing use of silver nanoparticles as antiseptics is related to the possibility that AgNPs are released into surface waters and, consequently, impact on aquatic organisms [92,93]. AgNPs can affect the ecosystem and nitrogen cycle and can also be transferred to the food web, thus posing a health risk [94].

Silver nanoparticles were tested on various model organisms, from single-celled organisms to organisms with a developed multiorgan system, against which they showed positive and negative effects in their toxicity [95]. Innovative approaches are also used to reduce/replace animal studies, such as reliable and predictive new approach methodologies (NAM), based on advanced in vitro methods (cytotoxicity and genotoxicity assays in cell lines) and in silico modelling (e.g., quantitative structure–activity relationships [QSAR]). 

The toxicity of nanoparticles varies depending on their concentration and size [96], surface charge [37], shape [97], method of synthesis [98], functionalisation of their surface [99], and time and route of administration, as well as the tested model or individuality of each organism [99,100]. The adverse effects of silver nanoparticles may appear in the form of mild irritation of the eyes and skin. AgNPs can also act as a skin allergen [101]. AgNPs are added to wound dressings as a disinfectant agent. They are also used in face creams and masks, and in clothing that reduces odours that come into contact with the skin [31,32]. Sensitisation tests on the skin of guinea pigs showed weak sensitisation to silver nanoparticles, with only 1 in 20 animals having discrete or patchy erythema [101,102].

Studies on the effects of silver nanoparticles on the skin of animals or on the eyes show no major irritation. The most common are small skin blushes and redness or spots on the eye [103,104,105]. In a 2010 study by Samberg et al., microscopic and ultrastructural examination revealed the occurrence of focal inflammation on the surface and in the upper stratum corneum layers of porcine skin after 14 days of topical application of AgNPs to the skin [32]. Alsaleh et al. assessed whether initial exposure of bone marrow-derived mast cells (BMMC) to 20 nm AgNP increased degranulation and allergen activation (human serum albumin conjugated to dinitrophenol). Research has shown that exposure to AgNPs has the potential to stimulate mast cells to allergic immune responses [106].

### 3.6. Organ Toxicity of AgNPs

AgNPs can be administered to the body through various routes, for example, inhalation; the first target organs are the lungs, where AgNPs can accumulate [89,90]. Oral exposure and skin contact are other ways of introducing silver nanoparticles into the body, as well as intraperitoneal (ip) or intravenous (iv) injections [107]. Once introduced into the body, the circulatory system is then responsible for distributing the nanoparticles that travel from the bloodstream to the rest of the body [82]. 

AgNPs can cause DNA damage, genotoxicity, an inflammatory response, and dysfunction in many organs, including the liver, kidneys, lungs, heart, and others [91].

Sung et al. studied the subchronic toxicity of silver nanoparticles in male and female Sprague–Dawley rats, with the animals exposed to silver nanoparticles with an average diameter of 18–19 nm for 6 h/day, 5 days/week, for 13 weeks in a full-body inhalation chamber. The target organs for the silver nanoparticles were the lungs and liver in both male and female rats. It was observed that liver bile duct hyperplasia increased dose-dependently in both male and female rats. Dose-dependent increases in lesions related to exposure to silver nanoparticles were also observed, such as mixed inflammatory cell infiltrate, chronic alveolar inflammation, and small granulomatous lesions [108]. Another study showed that nanoparticles can cause changes in the lungs and affect their normal functioning. After 90 days of inhalation exposure to AgNP at a dose of 2.9·10^6^ cm^−3^ particles, the rats developed lung inflammation and the tidal and minute volumes decreased significantly [109].

Garcés et al. evaluated the harmful effects developed in the lung after acute AgNP exposure. The studies were carried out in vivo on Balb/c mice, which were intranasally instilled with 0.1 mg AgNP/kg body weight. The lungs were the main collecting organ. with an increase in protein content and total cell counts observed in bronchoscopy and bronchoalveolar lavage (BAL) samples, resulting in barrier failure. An increase in active mitochondrial respiration and NOX activity was also observed, resulting in altered O_2_ consumption in lung tissue. This resulted in a review of reactive oxygen species (ROS) release that triggers the antioxidant system, observed by increased SOD, catalase and GPx activity, and a decrease in the GSH/GSSG ratio. Increased protein oxidation has also been demonstrated [110]. The liver is the second-most important target after the lungs for inhalation toxicity and the main target organ for oral toxicity [90]. In a study conducted in rats with 20–100 nm size intravenous administration of AgNP, the effect of systemic toxicity at 28 days was evaluated. In the presence of silver nanoparticles, a significant increase in the size of the spleen and an increase in the population of T and B cells were observed. AgNPs accumulated mainly in the spleen and liver but also in other organs. Liver damage has been demonstrated by determining increased phosphatase, alanine transaminase, and aspartate transaminase [111]. In a study conducted after 28 days of oral administration of silver nanoparticles, with or without polyvinylpyrrolidone (PVP) coating, to mice by gavage at a dose of 10–250 mg/kg of body weight per day, it was observed that both coated and uncoated AgNPs can cause subacute toxicity and oxidative damage in mice and accumulate primarily in the liver and spleen. AgNPs can be absorbed into the bloodstream, cross the blood–brain barrier, and be widely distributed in mice. Inflammatory changes in the lungs and liver were observed at high doses of both AgNPs [112]. In another study, in which 40 male Wistar rats were orally administered solutions containing 30, 125, 300, and 700 mg/kg AgNPs, after 28 days of exposure, histopathological changes were demonstrated, such as degenerative changes in the glomeruli, loss of tubular architecture, loss of brush border, and intermittent tubular basal lamellae that were more noticeable in the AgNPs’ 30 and 125 mg/kg groups [113]. Greater apoptosis was observed in the AgNPs’ 125 and 300 mg/kg groups. Gherkhbolagh et al., after a 28-day study of rats, observed a higher nephrotoxicity in the lower dose group (125 mg/kg) than in the higher dose group (300 mg/kg or higher) after oral administration of 250 nm AgNP [114]. Activation of cell proliferation and survival signalling pathways and release of pro-inflammatory cytokines were also observed after subchronic oral exposure (60 days) of female Wistar rats to 50 nm AgNPs at doses of 50 and 200 ppm, resulting in inhibition of the apoptotic pathway and necrotic cell deaths [115]. After 96 h of exposure to AgNPs with different surface coatings (sodium citrate and polyvinylpyrrolidone) at 20 nm and 100 nm in the gills, intestines, and muscles of zebrafish, the toxicity was tested. Citrate-coated AgNPs were more toxic than polyvinylpyrrolidone-coated AgNPs, and 20 nm AgNPs were more toxic than 100 nm AgNPs. The toxic effect of AgNPs in zebrafish tissue at the molecular level was confirmed by the differential expression of genes with different AgNPs [116].

Silver nanoparticles can enter the digestive system after ingestion or through the systemic circulation [117]. In the digestive system, nanoparticles mainly disturb the intestinal microflora, which can cause disorders similar to colitis, such as inflammatory and metabolic dysbiosis, which cause weight disorders [90]. After 21 days of oral administration of AgNP, ranging from 3 to 20 nm at doses of 5, 10, 15, and 20 mg/kg of body weight, the effect of AgNP on the mucosa of the small intestine was assessed. A significant decrease in body weight of mice was observed in all groups treated with AgNPs. At a dose of 10 mg/kg AgNPs, the mice showed maximum weight loss [76]. 

After contact of AgNPs with the cardiovascular system, histopathological changes, the release of pro-inflammatory cytokines, and oxidative stress, as well as the modification of cardiovascular parameters, can be induced [90]. For toxicological evaluation, male guinea pigs were exposed dermally to AgNP at concentrations of 100, 1000, and 10,000 ppm over a period of 13 weeks. In addition to the effects observed on the skin, kidneys, bones, and other organs, cardiomyocyte deformation was observed after administration of a higher dose of AgNP [118]. Holland et al. observed cardiovascular damage and an increased release of cytokines such as IL-2, IL-6, and IL-18 in rats after inhalation of a single dose of 200 μg/rat AgNP/PVP of 20 nm tested after 24 h [119]. 

Studies also show the neurotoxic effect of AgNPs after two weeks of exposure of rats to 10 nm AgNP at a dose of 0.2 mg/kg b.w. Induction of oxidative stress has been observed in myelin membranes, which can contribute to the disruption of myelin sheaths at the ultrastructural level [120]. Another study showed the neurotoxic effect of silver nanoparticles, where, after treating Wistar rats with 10 nm AgNP stabilised with citrate for 14 days at a dose of 0.2 mg/kg b.w., it was found that the animals had oxidative stress in the brain compared to the control group receiving saline [121]. Functional changes in the blood–brain barrier (BBB) were also observed in the brains of mice and rats, resulting in the formation of cerebral oedema after a single administration of silver nanoparticles (size 50–60 nm) intravenously at a dose of 30 mg/kg body weight or intraperitoneally at a dose of 50 mg/kg b.w. [122,123]. 

### 3.7. Toxicity Mechanisms

#### 3.7.1. Mechanism Related to Oxidative Stress

In vitro studies of the activity of silver nanoparticles using various types of cell models demonstrated the cytotoxicity of AgNP and allowed the understanding of the molecular mechanisms of their action [124]. The first steps in the transport of AgNPs inside the cell are the recognition, internalisation, and translocation of cell membrane receptors, and the last steps are degradation, accumulation, or removal by cells. AgNPs of small size (<5 nm) can passively penetrate cell walls and cell membranes. For most cells, internalisation of larger AgNPs occurs through endocytosis, with endosomes and lysosomes being the major target organelles [3,125]. Most studies agree that the induction of oxidative stress and production of reactive oxygen species (ROS) determine the cytotoxicity of silver nanoparticles. The mechanism proposed by Park et al. explains this in such a way that AgNPs act as carriers to transfer silver across the cell membrane, and in the intracellular environment, AgNPs dissolve to release Ag^+^, resulting in the production of reactive oxygen species. Dissolved silver cations cause oxidative stress which is considered one of the most important toxicity mechanisms associated with exposure to silver nanoparticles. ROS appear in the cell as by-products of biological oxygen metabolism but are kept at a low level by the antioxidant defence mechanisms of the cell and do not affect the normal physiological activity of cells [69]. Under oxidative stress, there is a depletion of glutathione and other antioxidants and an increase in ROS production, which leads to an imbalance between ROS production and its neutralisation by antioxidant enzymes and antioxidants [3,124]. Interactions of cellular macromolecules and genomes with ROS can alter their normal activity. There is damage to genetic material and induction of genes related to apoptosis [126]. 

Some authors propose that AgNPs themselves induce the formation of reactive oxygen species and free radicals, which, for example, in the case of bacteria, causes damage to intracellular organelles and modulation of intracellular signalling pathways resulting in apoptotic cell death [107]. In a study conducted by Massarsky et al., Ag^+^ and AgNP were shown to increase ROS production [127].

Silver nanoparticles also show cytotoxic effects on cancer cells, acting as antitumour agents and slowing the growth of cancer cells. This may be due to the inhibition or activation of many signal pathways associated with autophagy, mitochondrial dysfunction, reactive oxygen species production causing oxidative stress, and endoplasmic reticulum stress. Silver nanoparticles are also responsible for the aetiology and development of cancer [128,129]. 

#### 3.7.2. Mechanism Related to Non-Oxidative Stress

Most studies on the toxicity of nanoparticles have been explained by the release of metal ions [130]. One of the mechanisms not dependent on oxidative stress in the case of bacteria may be the release of Ag^+^ ions, after the interaction of AgNPs with the bacterial cell, which may encounter sulphur-containing proteins in the cell wall, affecting proper functionality [131]. Another proposed mechanism of bacterial cytotoxicity is the adhesion of silver nanoparticles to the bacterial wall, followed by infiltration of particles and damage to the bacterial cell membrane, leading to the leakage of cellular contents and death [107]. Wu et al. (2018) found that these two types of mechanisms are responsible for the antimicrobial effect of silver nanoparticles, i.e., the contact of AgNPs with the cell and the release of silver ions [132]. 

AgNPs and silver ions can interact with proteins and amino acids, with this believed to be an important mechanism of silver nanoparticle toxicity. Saptarshi et al. suggested that AgNPs can cause protein crown formation, protein unfolding, and protein dysfunction [133]. Studies have also shown that mitochondria are targeted by released silver ions and that they readily undergo the permeability pathway, leading to the formation of protein pores in the mitochondrial membranes. In rats, increased permeability in liver mitochondria caused mitochondrial swelling, abnormal metabolism, and led to apoptosis [134]. 

AgNPs can also interfere with autophagy. They cause abnormalities in autophagosome–lysosome fusion, which consequently leads to abnormal accumulation of enlarged autophagosomes in the cytosol and exacerbation of AgNP-induced cytotoxic consequences such as DNA damage, mitochondrial impairment, and cell death [135].

Silver nanoparticles can interact directly with DNA, changing its conformation and causing damage to it. Direct interaction of AgNPs with DNA may be an inducer of genotoxicity [136].

The exact mechanism of silver nanoparticle toxicity is unknown, especially because it depends on many properties, such as the interaction of silver nanoparticles with other particles, drugs, and contaminants, and can reduce or improve toxicity. Nanoparticles rarely exist alone in the environment, and this is another topic covered in this paper.

### 3.8. Complex Toxicity Evaluation of AgNPs

The increased use of silver nanoparticles in our daily lives and in industry has increased the vulnerability of ecosystems [137]. The presence of these nanostructures in the environment is also associated with their natural formation, for example, during mining, or as a result of extracellular or intracellular microorganisms synthesis [138]. AgNPs, after being released into the environment, can undergo various transformations such as aggregation, agglomeration, or dissolution, after which various chemical compounds are formed (e.g., sulphides or chlorides) [91]. Another transformation of AgNPs in the environment is their combination with various substances, which are mainly natural organic material. On the basis of the composition of the organic material and the capping agent of the nanomaterial, the solubility and aggregation of the nanoparticles can be changed. For example, the aggregation of AgNPs may be reduced when AgNPs interact with organic material to form a coating that stabilises the nanoparticles. Organic compounds found in the environment can also form silver nanoparticles by reducing silver ions [139]. Therefore, nanoparticles in the environment do not occur alone, but in combination with various chemical compounds or undergo transformations, which is why their complex toxicity after such transformations should be investigated [25]. 

The toxicity of metallic nanoparticles for aquatic organisms is related to their physical and chemical properties, as well as to their transformations that take place in the environment, such as aggregation, dissolution, or agglomeration [130]. The presence of divalent cations (e.g., Ca^2+^ and Mg^2+^) destabilises silver nanoparticles. Increasing the ionic strength causes weakening of the electrostatic repulsion processes between the particles and reduction of the double electric layer on the surface of the silver nanoparticle, leading to the phenomenon of aggregation [137]. The size and composition of the silver nanoparticle’s coating play a key role in its aggregation and chemical transformation in the environment [140]. AgNPs are often used as antimicrobial agents, but the toxicity of these nanomaterials to aquatic organisms, algae, plants, fungi, and (in)vertebrates has also been observed [141]. As we already know, small nanoparticles often have a greater antibacterial effect. There are many microorganisms in the environment that play a key role, so it is important to maintain balance in the ecosystem. Silver nanoparticles disturb this balance, exerting a toxic effect on these microorganisms [142]. Coating also influences the transformation of AgNPs in the environment; for example, greater stability and less size changes were observed for AgNPs coated with polyethylene glycol (PEG) and polyvinylpyrrolidone (PVP) compared to AgNPs coated with citrate [143]. The determination of the possible ways of transformation of silver nanoparticles and the parameters that affect it is crucial in the assessment of the complex toxicity of AgNPs.

In the environment, silver nanoparticles undergo severe transformations, such as interactions with other compounds that change toxicity mechanisms and make them more complex. Further studies are needed on this issue.

## 4. Safety Assessment of Silver Nanoparticles in Cosmetic Products

The European Commission (EC) received notifications (*n* = 63) for cosmetic products containing nanoform colloid silver (CAS 7440-22-4, EC 231-131-3). This important ingredient is reported in the CosIng database, which does not mention nanoforms with the functions of abrasion, emulsion, and stabilisation of emulsion, but is not regulated by Cosmetics Regulation (EC) No. 1223/2009. The CE advisory body for cosmetic product safety assessment is the Scientific Committee on Consumer Safety (SCCS). SCCS provides opinions on the health and safety risks (chemical, biological, mechanical, and other physical risks) of non-food consumer products (such as cosmetics and their ingredients, toys, textiles, clothing, personal care products, and household products) and services (such as tattoos and artificial sunbathing). The initial evaluation of all data submitted showed that the data provided by the applicants appeared to be very minimal. There were no complete dossiers for all colloid silver dispersions and no data on physical and chemical characteristics and experimental toxicological studies. Clarification of certain aspects and a lack of safety data are necessary before the SCCS can formulate an opinion on the safety of the material. To facilitate the evaluation process, applicants were asked to provide additional data, amend the information submitted to the dossier, and provide clarification. Some applicants provided additional data and information. All relevant information on colloidal silver dispersion, including additional data, has been described and assessed in this document. Furthermore, the Commission has issued an information appeal on the safety of nanosilver. Information obtained from various sources was also taken into account in this opinion. According to the applicants, nanoform colloid silver is used in the form of nanoelectronic coatings in oral cosmetic products that are left on and cleaned, including toothpaste and skin care products with a maximum concentration limit of 1% reported and specifications as indicated in the list attached to the mandate. It should be noted that the applicants provided only limited data corresponding to the SCCS Safety Assessment Guidelines (SCCS 1484/12). The data provided did not correspond to the SCCS Memorandum on the Relevance, Accuracy, and Quality of Data in Safety Dossiers for Nanomaterials (SCCS/1524/13). As a result of several large data gaps, SCCS cannot draw conclusions about the safety of nanocolloid silver used in oral and skin cosmetics. Therefore, the safety assessment of silver nanoparticles in cosmetic products remains an open topic in the cosmetic industry.

## 5. Green Toxicology of Silver Nanoparticles

### 5.1. Green Synthesis of Silver Nanoparticles

**Figure 1:**
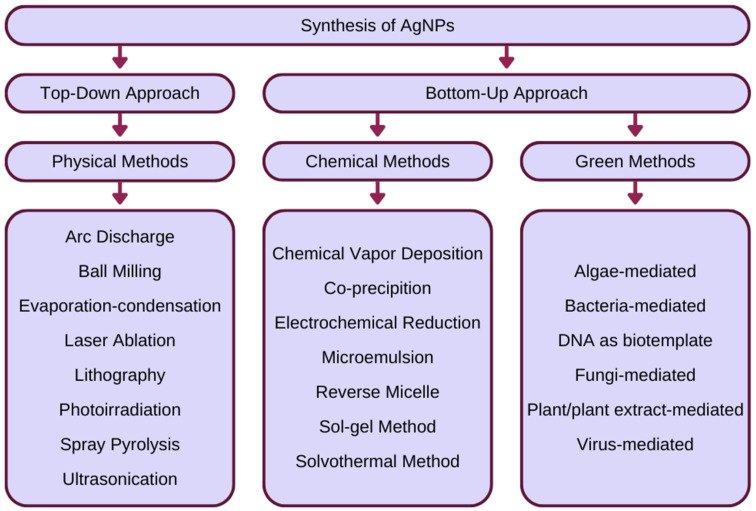
Classification of physical, chemical, and green synthesis techniques of AgNPs, based on [139,144].

The green synthesis of nanoparticles can be characterised as a set of procedures that use non-hazardous methods or non-chemical reagents for the production of nanoparticles. The main purpose of this technique is to minimise environmental toxicity and health threats [144,145]. To overcome this problem, scientists discovered exact pathways using naturally occurring sources and their products that can be used to synthesise nanoparticles [143,146]. Biological methods are extremely valuable as they utilise natural processes to synthesise nanoparticles. In the 19th century, scientists discovered the reduction ability of biological materials, laying the foundations for future green synthesis [147]. Most of the processes of synthesis of green silver nanostructures involve the reduction of Ag^+^ to Ag^0^, performed by biological species or by compounds of biological origin of the appropriate type of plant or organism [145,148]. Green synthesis, as with the chemical method, is classified as a bottom-up approach, in contrast to the physical method classified as a top-down approach [144]. The classification and examples of silver nanoparticle (AgNP) synthesis techniques is illustrated in Figure 1.

The synthesis of AgNPs is also possible by using laser irradiation (which does not require any reducing agent), microwave irradiation, ionising irradiation, and pulse radiolysis [145,148]. These techniques may also be classified as green synthesis methodologies because they use harmless procedures to produce nanoparticles. However, their disadvantage is high-level energy consumption [149]. Biological methods have several advantages over physical and chemical methods. First, they are environmentally friendly as opposed to chemical methods [150]. They consume less energy compared to physical methods and therefore are energy efficient [145]. They are economically viable, being used for mass production [151]. Their renewable nature provides a wide variety of reagents that can act as reducing agents, and therefore is a method [152]. In addition to the advantages mentioned above, the process is also efficient. A high synthesis of AgNPs was achieved using an aqueous extract of *Artemisia absinthium* (90%) [153]. Using *Chlorella vulgaris* extracts, the synthesis resulted in AgNPs with low polydispersity and good yield (>55%) [148]. Of particular importance in the green synthesis of AgNPs is the optimisation of the technique in terms of scalability, product quality, and efficiency. The reaction conditions can be improved by changing the pH, temperature, redox conditions, incubation period, and salt concentration [139]. For example, pH can affect the size of nanoparticles; in plants, changes in pH lead to changes in the charge of phytochemicals, which affect the reduction and binding of Ag in the synthesis process [151,154]. Another important aspect is the selection of an appropriate method for extracting nanoparticles from plants and microorganisms. Physicochemical methods useful for this purpose include freeze-thaw, heating, osmotic shock, and enzymatic lysis (expensive, therefore not suitable for an industrial scale). These methods can modify the structure, size, aggregation, and shape of nanoparticles [151].

### 5.2. Biotemplates Used for the Green Synthesis of Silver Nanoparticles

The synthesis of AgNPs using marine microorganisms has proven to be a promising method because of its non-toxic and environmentally friendly nature. The most common types of algae used for the synthesis of AgNPs are cyanobacteria, brown algae, and green algae [155]. The composition of homogeneous microalgae suspensions in the form of biomass in the aqueous phase, cell-free aqueous extract, or an aqueous supernatant of dried algae can be used directly in the AgNPs’ synthesis process by mixing with a silver solution (mainly silver nitrate) [155]. Algae-mediated synthesis can be intracellular as well as extracellular, depending on the type of cell culture used [156]. Wall-deficient cells are also prone to intracellular biosynthesis because the cell wall acts as a barrier to the diffusion of metal cations into the cytoplasm [157]. Once synthesised, AgNPs are covered by a matrix of polysaccharides that enter and exit cells [156]. Silver nanoparticles showed effective antioxidant and antibacterial activity [158]. Silver nanoparticles produced by green synthesis can be characterised by the following techniques: scanning electron microscopy (SEM) and transmission electron microscopy (TEM) [136,139]. The advantages of the synthesis of AgNPs by algae are low reaction temperatures, the use of harmless reagents, and the synthesis of relatively small particles with a uniform morphology. However, the disadvantages include a significantly low production rate [158]. *Chaetoceros calcitrans*, *Chlorella salina*, *Isochrysis galbana*, and *Tetraselmis gracilis* produce metabolites that can reduce silver ions and therefore synthesise AgNP [156,159]. The proteins contained in the *Chorella vulgaris extract* reduce Ag^+^ ions and synthesise nanoparticles at room temperature in a controlled form: Ag nanoplates with an average length of 44 nm and a width of 16–24 nm [160].

Bacteria produce many extracellular and intracellular inorganic materials, making these microorganisms an efficient biofactory. Gram-positive and Gram-negative strains can be used for the non-enzymatic production of AgNPs through the interaction of silver ions with organic compounds present in bacterial cells [136,139]. Silver nanoparticles are known for their toxic properties to bacteria; therefore, to enable their synthesis, they must be made resistant to nanoparticles by incorporating the “sil” gene with plasmids [161]. One of the AgNPs’ synthesis approaches is the usage of bacterial biomass and AgNO_3_ solution under appropriate temperature and pressure conditions [162]. Another approach to AgNPs’ bacteria-mediated green synthesis is the bioreduction route. The reaction takes place both intracellularly and extracellularly, depending on the site of the reduction of silver ions [163]. Ag^+^ are reduced to AgNPs by the reductase enzyme, NADH-dependent, from which it takes electrons [164]. The disadvantage of the bacterial-based green synthesis method is the limited spectrum of sizes and shapes obtained [144]. The isolated strain of *Pseudomonas stutzeri AG259* found in the silver mine is the first evidence of bacterial production of AgNP [162,165]. *Spirulina platensis* is also utilised for the extracellular synthesis of nanoparticles. AgNPs with a size of 7–16 nm are obtained under optimal conditions: 37 °C, 120 h, and pH 5.6 [166]. *Plectonema boryanum* precipitates spherical AgNPs with a size of 200 nm, while *Bacillus subtilis* produces AgNP with a size of 5–60 nm by applying microwave radiation [167]. The synthesis of spherical AgNPs with an average diameter of 10–12 nm is also possible with *Rhodococcus* spp., during 10 h of incubation at room temperature [168]. The *Corynebacterium SH09* strain produces silver nanoparticles of a size of 10–15 nm by bioreduction of diamine silver complexes [167]. *Lactobacillus*, *Enterococcus*, *Pediococcus pentosaceus,* and *Enterococcus faecium* reduce silver ions under alkaline conditions [160].

Fungi-mediated green synthesis has shown a promising approach for the production of AgNPs; the less pathogenic behaviour of fungi and their faster synthesis rate suggest their advantage compared to bacteria [149]. Fungi secrete much higher amounts of bioactive substances than bacteria; therefore, fungi are believed to be more suitable for large-scale production [169]. In addition, the advantages of using fungi as a biofactory include the production of nanoparticles of various sizes and chemical composition in a monodisperse manner [163]. Fungi not exposed to high concentrations of toxic metals have the innate ability to produce higher concentrations of proteins that reduce metals [170]. The synthesis of silver nanoparticles by fungi can take place both intracellularly and extracellularly [171]. The reduction of Ag^+^ ions occurs through cell wall polymers or electron shuttle quinones that act as redox centres. NADPH-dependent nitrate reductase reduces Ag^+^ ions. Nitrogen peptides or biomacromolecules stabilise AgNPs [169,172]. AgNPs synthesised with fungi have been shown to have noticeable antibacterial activity. Synthesised AgNPs using *A. flavus* fungi significantly increased biocidal efficacy against drug-resistant bacteria [170,173]. AgNPs with an average diameter ranging from 1 to 10 nm were synthesised in culture supernatants of *Aspergillus terreus;* this synthesis was mediated by an extracellular enzyme [171,174]. *Fusarium oxysporum* synthesises silver nanoparticles extracellularly by reducing silver nitrate. The fungus strain secretes the AgNPs’ stabilising protein and the silver ions are reduced by nitrate-dependent reductase [175].

There are increasing numbers of studies using plant extracts as reducing, protecting, and stabilising agents for the synthesis of AgNP [173,176]. The synthesis of silver nanoparticles based on plants and their extracts is non-pathogenic, simple, one-step, and additionally has a higher bioreductive potential compared to the synthesis from microbial culture [174,177]. The availability of the reducing agent is more concentrated in the extract than in the whole plant. The synthesis of silver nanoparticles from green plants consists of mixing a plant extract with an aqueous solution of the metal salts [176]. This works by reduction mediated by phytochemicals: terpenoids, flavones, ketones, aldehydes, amines, and carboxylic acids. Flavones, organic acids, and quinones are involved in the immediate reduction of Ag^+^ ions [165]. AgNPs with a spherical shape and size in the range of 22.3–48.2 nm were synthesised using the *Odontosoria Chinensis* extract. The complete reduction of Ag^+^ ions occurred after 10 min at 40 °C. The synthesised AgNPs showed effective anti-inflammatory and antidiabetic activity, which was the result of the presence of polyphenols, terpenoids, and tannins in the extract, in addition to AgNP itself [175,178]. The amine groups present in the *Capsicum annum* proteins act as controlling and reducing agents for the formation of silver nanoparticles [179]. AgNPs with an average size of 9–10 nm were produced with aqueous extract of *Parthenium hysterophorus*. The aqueous extract was mixed with the AgNO_3_ solution, and the mixture was incubated in the dark for 1 h. In addition to significant antibacterial activity, the synthesised AgNPs also showed antitumour activity against HepG2 cell lines [177,180]. The biomass of *Cinnamonomum camphora* leaves treated with aqueous silver precursors at ambient temperature produces AgNPs with a circular shape. The protective and reducing biomolecules present in the plant are responsible for the shape of the nanoparticles [178,181]. Green synthesis using *Mentha aquatica* leaf extract produces small AgNPs. The most optimal reaction conditions are: temperature 90 °C, reaction time 60 min, and pH 9.5 [182]. Mixing the silver nitrate solution with the *Nelumbo nucifera* extract results in the formation of silver nanoparticles [183]. Spherical AgNPs were generated using the bark extract of *Picea abies L.* The size distribution of the resulting nanoparticles was significantly high (100–500 nm). Ag^+^ ions were reduced for 3 h at 70 °C and pH 9 [184].

In recent years, viruses have been used as biotemplates for the synthesis of nanoparticles. They are rarely used compared to other methods of green synthesis. AgNPs can be synthesised inside the viral template, within the interface, or on the outer surface [182,185]. The Tobacco Mosaic Virus (TMV) is used as the most common biotemplate for the production of rod-shaped silver nanoparticles. AgNPs were coated on the inner surface of the TMV channel. Amino acid functional groups mediate the synthesis process [186]. In addition to TMV as biotemplates, the following can be used for the synthesis of silver nanoparticles: Turnip Yellow Mosaic Virus (TYMV), Hibiscus Chlorotic Ringspot Virus (HCRSV), Red Clover Necrotic Mosaic Virus (RCNMV), Brome Mosaic Virus (BMV), and Cowpea Mosaic Virus (CPMV) [185]. An advantage associated with the usage of viral matrices is the simple synthesis of small AgNPs. However, their disadvantage is the lack of strong metal binding sites along the surface of the biotemplate [184,187]. Moreover, the preparation of viral templates is time consuming and multiple coating cycles may be required to obtain a homogeneous coating of metal nanostructures on their surface [149].

DNA also serves as a template for the synthesis of nanoparticles. The nucleoprotein filament from the polymerisation of RecA proteins on a single-stranded DNA probe was mixed with a long double-stranded DNA substrate derived from an aldehyde. The sample was exposed to AgNO_3_ ions that bound to dsDNA in the absence of RecA, and the aldehyde groups reduced the Ag^+^ species to Ag^0^ and formed silver nanoparticles [188].

A summary of the methods of green synthesis of AgNPs is presented in Table 1.

### 5.3. Applications of Green Synthesised Silver Nanoparticles

Silver nanoparticles have numerous applications that have revolutionised applied medicine, mainly due to their antimicrobial and antifungal properties. AgNPs have been widely used as wound dressings and creams or as an antibacterial coating in therapeutic applications such as cardiovascular implants, catheters, dental composites, and nanobiosensing [194]. Silver nanoparticle wound dressings are used in the clinical treatment of various injuries such as burns and chronic ulcers [195]. These wound dressings, compared to the gauze dressing or cream previously used, containing 1% Ag, significantly reduced the therapeutic time of the injury while increasing the bacterial clearance of contaminated injuries [178,181]. Chitin-AgNPs used in wound dressings had antibacterial potential in wound therapeutic applications [196]. The silver-coated silicone heart valve induced an allergic reaction and inhibited the proper functioning of the fibroblasts in the patient; therefore, it was decided to use AgNP instead of the Ag element [197]. Silver nanoparticles proved to be a safer, non-toxic, and antibacterial surface coating of heart valves and stents. Furthermore, the introduction of nanoparticles into the core of the polymer backbone in heart valves increases their biocompatibility and resistance to calcification [198]. The physicochemical properties of silver at the nanoscale resulted in the improvement of biosensors and the development of nanobiosensors, which are used in disease diagnosis, therapy monitoring, cell tracking, and in vivo detection of nanoprobes [199]. In a standard hospital setting, catheters are highly prone to contamination. The use of AgNP coating reduced the development of biofilms in catheters, effectively reducing the number of bacteria to 72 h, and was non-toxic [200]. In the textile industry, silver non-toxic nanoparticles possessing antimicrobial characteristics are used to create sterile hospital clothing that counteracts or minimises contamination with pathogenic bacteria such as *S. aureus* [198,201]. AgNPs have also found an application in dentistry. For example, as a component of orthodontic adhesive, AgNPs increase resistance to bacteria, improving the bond strength of the orthodontic adhesive [199,202] and the coating of dental instruments, which reduces microbial colonisation and improves the antifungal efficacy [200,203]. AgNPs synthesised by plant extracts show an antidiabetic potential. Silver nanoparticles synthesised using *Solanum nigrum* leaf extract reduced blood glucose levels in alloxan-induced diabetic rats. Compared to the standard antidiabetic drug (glibenclamide), AgNPs showed a hypoglycaemic effect [204]. In addition, silver nanoparticles synthesised with *Argyreia nervosa* leaf extract showed antidiabetic activity. They inhibited the action of two digestive enzymes, α-amylase and α-glucosidase [205]. AgNPs are also present in consumer products, including water filters, deodorants, soaps, socks, and room sprays [206]. In general, there has been a great deal of effort to develop green synthesis in the past few years. Green synthesis is ahead of chemical and physical methods because it is cost effective, environmentally friendly, non-toxic, and effectively scaled up for large-scale synthesis. The growing awareness of the use of green synthesis to produce metal nanoparticles, especially AgNPs, has led to the use of these nanoparticles in nanotechnology and, at the same time, abundant practical applications.

## 6. Conclusions

The toxic effect of silver nanoparticles is desirable in modern anticancer treatments, as a new strategy for curing antibiotic-resistant bacterial infections, and in other diseases. Targeted therapies are studied to avoid destroying healthy cells. New possibilities are emerging for the synthesis of non-toxic AgNPs and those with toxic properties when needed. In recent years, the number of applications and the amount of nanosilver used has increased significantly. AgNPs have been shown to inhibit the growth and survival of bacteria, including human and animal pathogens, and they have antiviral and anticancer properties. They also inhibit the development of bacterial biofilms and therefore may be a promising alternative to conventional antibiotics. Currently, they are the most commercialised metal nanoparticles and are included in numerous products, such as antibacterial dressings, home water treatment plants, textiles, and cosmetics. The development of methods for environmentally safe “green synthesis” of silver nanoparticles would increase the safer production and biological use of nanosilver. 

Most studies have focused on the therapeutic targets of AgNPs, but whether AgNPs may be hazardous to many systems, such as the skin, eyes, kidneys, respiratory, hepatobiliary, and immunological, has also been discussed. More detailed research should be undertaken to evaluate the biocompatibility and potential cytotoxicity of AgNPs, which could aid in the creation of more secure and biocompatible agents based on AgNPs. In addition, an in vitro-to-in vivo extrapolation is required to support the development of the next generation risk assessment (NGRA) strategy for AgNPs [207].

## Figures and Tables

**Table 1:** Green synthesis methods of silver nanostructures: comparison and classification [144,156,158,186,187,189,190,191,192,193].

Method	Advantages	Disadvantages	Reference
algae-mediated synthesis	• simplicity • low cost • environmentally friendly • uniform morphology of nanoparticles • usage of non-pathogenic and non-hazardous reagents • small size of nanoparticles	• slow synthesis rate • unknown biological functions that affect synthesis	[156,158]
bacterial-mediated synthesis	• simplicity • environmentally friendly	• pathogenic behaviour of species such as *E. coli* • slow synthesis rate • large size distribution • unknown biological functions that affect synthesis	[144,189]
fungi-mediated synthesis	• simplicity • environmentally friendly • fast synthesis rate • high bioaccumulation capacity • high intracellular uptake • usage of non-pathogenic reagents	• longevity of process • pathogenic behaviour • unknown biological functions that affect synthesis	[190,191]
plant-mediated synthesis	• simplicity • low cost • environmentally friendly • broad scope • low reaction temperatures • usage of non-pathogenic and non-hazardous reagents • act as both reducing and capping agent at the same time	• unknown mechanisms that affect synthesis	[192,193]
virus-mediated synthesis	• simplicity • environmentally friendly • small size of nanoparticles	• time-consuming preparation of the biotemplate • required multiple coating cycles to yield a uniform coating	[186,187]

## Data Availability

The data, analytic methods, and study materials that support the findings of this study are available from Kamil Jurowski (kjurowski@ur.edu.pl) on reasonable request.

## Abbreviations

AgNPs—silver nanoparticles; BAL—bronchoscopy and bronchoalveolar lavage; BBB—blood–brain barrier; BMMCs—bone marrow-derived mast cells; BMV—Brome Mosaic Virus; CPMV—Cowpea Mosaic Virus; Cr—chromium; EBM—Evidence-Based Medicine; EC—the European Commission; EI—elemental impurities; ESCs—embryonic stem cells; ET AAS—electrothermal atomisation atomic absorption spectrometry; GSH—glutathione; HCRSV—Hibiscus Chlorotic Ringspot Virus; HHRA—human health risk assessment; HUVEC—human umbilical vein endothelial cells; ICH Q3D—International Council for Harmonisation of Technical Requirements for Pharmaceuticals for Human Use; MET—transmission electron microscopy; MoA—mode of action; MoE—margin of exposure; NAMs—new approach methodologies; PBMCs—peripheral blood mononuclear cells; PDE—permitted daily exposure; PEG—polyethylene glycol; PoD—point of departure; PVP—polyvinylpyrrolidone; QSAR—quantitative structure–activity relationships; RCNMV—Red Clover Necrotic Mosaic Virus; REACH—European Regulation on Chemical Substances; ROS—reactive oxygen species; SCCS—Scientific Committee on Consumer Safety; SEM—scanning electron microscopy; TMV—Tobacco Mosaic Virus; TYMV—Turnip Yellow Mosaic Virus; WCS—worst-case scenario.
